# Pilot Study on Dynamic Long-Axial Field-of-View [^18^F]FDG PET/CT in Liver Transplant Recipients as a Non-Invasive Alternative to Routine Biopsies

**DOI:** 10.3390/diagnostics16071021

**Published:** 2026-03-28

**Authors:** Martin Bloch, Susanne Dam Nielsen, Barbara Malene Fischer, Allan Rasmussen, Hans-Christian Pommergaard, Flemming Littrup Andersen, Gro Linno Willemoe, Thomas Lund Andersen, Per Karkov Cramon

**Affiliations:** 1 Department of Clinical Physiology and Nuclear Medicine, Rigshospitalet, 2100 Copenhagen, Denmark; barbara.malene.fischer@regionh.dk (B.M.F.); flemming.andersen@regionh.dk (F.L.A.); thomas.lund.andersen@regionh.dk (T.L.A.); per.cramon@regionh.dk (P.K.C.); 2 Department of Digestive Diseases, Transplantation and General Surgery, Rigshospitalet, 2100 Copenhagen, Denmarkhans-christian.pommergaard@regionh.dk (H.-C.P.); 3 Department of Infectious Diseases, Rigshospitalet, 2100 Copenhagen, Denmark; 4 Department of Clinical Medicine, Faculty of Health and Medical Sciences, University of Copenhagen, 2100 Copenhagen, Denmark; 5 Hepatic Malignancy Surgical Research Unit (HEPSURU), Rigshospitalet, 2100 Copenhagen, Denmark; 6 Department of Pathology, Rigshospitalet, 2100 Copenhagen, Denmark; gro.linno.willemoe.02@regionh.dk

**Keywords:** long-axial field-of-view [^18^F]FDG PET/CT, hepatic [^18^F]FDG kinetics, liver transplantation, allograft inflammation

## Abstract

**Background/Objectives**: Routine liver biopsies play an important role in monitoring liver allografts but carry non-negligible risks. This pilot study assesses the feasibility of dynamic long-axial field-of-view (LAFOV) [^18^F]FDG PET/CT as a non-invasive alternative to biopsy. **Methods**: Liver transplant (LTx) recipients meeting the inclusion criteria of ≥10 months post-transplantation and scheduled routine biopsy were prospectively enrolled, along with healthy controls. All participants underwent dynamic LAFOV [^18^F]FDG PET/CT, followed by biopsy in LTx recipients, who were stratified by inflammatory severity using the BANFF score. Hepatic kinetic parameters (K1, k2, k3, k4) and SUVmean/SUVmax were compared using Mann–Whitney U tests. Correlations were assessed using Spearman’s rank correlation. A *p*-value < 0.05 was considered significant. Analyses were performed in RStudio (version 2024.12.10563). **Results**: Sixteen LTx recipients (mean age 48.6 years; seven female, nine male) and eight healthy controls (mean age 35.4 years; six female, two male) were included. Healthy controls had mean k3 and k4 values of 0.0037 min^−1^ ± 0.0003 min^−1^ and 0.0019 min^−1^ ± 0.0011 min^−1^, respectively. LTx recipients showed significantly higher k3 and k4 values, both when including and excluding patients with biopsy-confirmed inflammation. Descriptive comparisons between LTx recipients with and without significant inflammation (*n* = 3) showed no clear differences. Spearman analysis showed no significant correlations between the BANFF score and kinetic parameters. The strongest degree of correlation was found between BANFF score and k3, indicating a moderate positive but non-significant association (k3: rs = 0.396, *p* = 0.128). **Conclusions**: Elevated k3 and k4 values in LTx recipients were not explained by allograft inflammation, suggesting altered FDG kinetics post-transplant. These differences may confound [^18^F]FDG PET interpretation. Larger studies are needed to assess the clinical applicability of dynamic LAFOV [^18^F]FDG PET/CT.

## 1. Introduction

### 1.1. Regarding the Need for Non-Invasive Tests for Graft Function

Liver disease is an increasingly prevalent cause of morbidity and mortality globally, accounting for one in every 25 deaths worldwide [1]. Many potentially deadly liver diseases, including end-stage cirrhosis, hepatocellular carcinoma, hepatitis, and advanced biliary disease, are most effectively treated with liver transplantation. However, liver transplantation is not a simple treatment, requiring specialized surgical skills and intensive postoperative immunosuppressive treatment [2,3].

Advancements in surgery and immunosuppression have improved life expectancy for liver transplant (LTx) recipients. Five-year survival rates have reached approximately 75% for adult recipients, and as high as 81% for children, with specific subgroups surviving even longer [3].

To prevent graft rejection, LTx recipients undergo lifelong treatment using a range of immunosuppressants. While highly potent and invaluable in preventing or delaying organ rejection, these immunosuppressants come with a range of serious long-term adverse effects, ranging from chronic nephrotoxicity to increased rates of infections and cancer [4,5]. To maintain the balance between minimizing adverse effects while preventing allograft rejection, the lowest possible effective dose of immunosuppression following strict immunosuppressant regimens is preferred [6,7]. To monitor the condition of liver allografts, routine biopsies can be used to detect inflammation and fibrosis, which indicate early signs of potentially reversible conditions that might otherwise compromise graft function, such as acute cellular rejection or early chronic cellular rejection [8,9].

However, a biopsy is an invasive procedure associated with non-negligible risks: studies have reported major complications (including bleeding and infections) in almost 2% of post-transplant liver biopsies, and mortality rates up to 0.2% [8,10]. To prevent this while enabling closer monitoring, the development of non-invasive tests for liver graft function is a priority.

### 1.2. [^18^F]FDG PET/CT Assessment of Graft Dysfunction in Liver Transplant Recipients as an Alternative to Biopsy

One non-invasive test that has shown potential to diagnose liver pathology in LTx recipients is 2-[^18^F]fluoro-2-deoxy-D-glucose ([^18^F]FDG) Positron Emission Tomography/Computed Tomography (PET/CT). Several human and animal studies using PET/CT have demonstrated increased hepatic uptake of FDG in individuals with confirmed acute liver rejection [11,12].

The application of conventional [^18^F]FDG PET/CT for evaluating inflammation in liver allografts faces several significant limitations. Most importantly, humans have inherently high physiological liver FDG uptake, which compromises diagnostic accuracy and clinical utility [13]. To overcome this limitation, dynamic [^18^F]FDG acquisitions can be utilized. Dynamic scans, in which data acquisition starts immediately upon [^18^F]FDG injection, enable the extraction of time–activity curves that can be quantitatively analyzed using established compartment modelling techniques. Specifically, compartment models can be used in the context of intrahepatic FDG kinetics [13].

**Figure 1:**
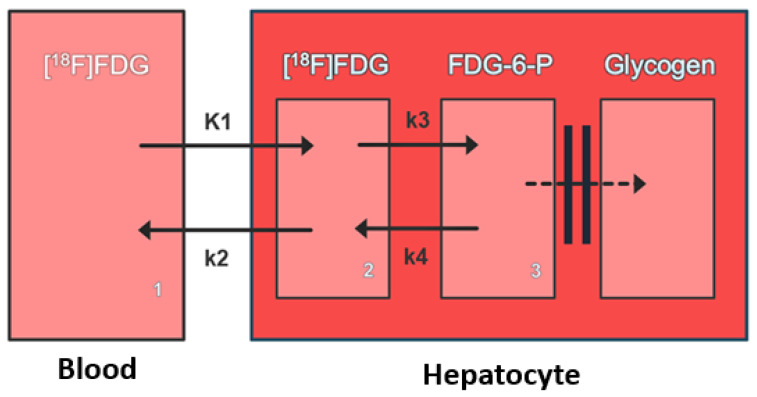
Three-compartmental model of intrahepatic [^18^F]FDG kinetics. K1 represents the unidirectional transfer of [^18^F]FDG from blood into hepatocytes. k2 represents the reverse efflux of unphosphorylated [^18^F]FDG from hepatocytes to the systemic circulation. k3 represents the phosphorylation and subsequent metabolic trapping of [^18^F]FDG within hepatocytes. k4 represents the dephosphorylation of [^18^F]FDG-6-phospate back to free [^18^F]FDG. The three compartments are 1: extracellular [^18^F]FDG, 2: intracellular [^18^F]FDG, and 3: intracellular FDG-6-P.

The 2-tissue compartment model of hepatic [^18^F]FDG metabolism is characterized by four kinetic rate constants (see Figure 1 below). K1 represents the unidirectional transfer of [^18^F]FDG from blood into hepatocytes, while k2 describes the reverse efflux of unphosphorylated [^18^F]FDG from hepatocytes to the systemic circulation. The rate constant k3 quantifies the phosphorylation and subsequent metabolic trapping of [^18^F]FDG within hepatocytes, whereas k4 represents the dephosphorylation of [^18^F] FDG-6-phosphate back to free [^18^F]FDG.

Previous investigations have suggested k3 as a potential biomarker for hepatic inflammation, although achieving statistical significance has been challenging using standard short-axial field-of-view (SAFOV) PET systems, citing image noise as the primary cause [14,15].

Long-axial field-of-view (LAFOV) scanners with enhanced sensitivity and improved count statistics may address this challenge by providing more reliable estimates of k3 (phosphorylation rate) and k4 (dephosphorylation rate), thereby improving the detectability of hepatic inflammatory processes [14]. Additionally, LAFOV PET systems have much lower radiation exposure than conventional scanners due to higher sensitivity and shorter scans [16,17,18,19]. This makes them especially advantageous for non-oncologic evaluations and vulnerable patients who may require repeated imaging [16,20,21].

### 1.3. The Aim of This Study

We hypothesized that dynamic [^18^F]FDG PET/CT can detect and quantify hepatic inflammation in LTx recipients by measuring alterations in intrahepatic FDG kinetics. We expected that LTx recipients with biopsy-confirmed allograft inflammation would exhibit significantly altered FDG kinetic parameters compared to those with histologically healthy grafts. More specifically, we expected that the phosphorylation rate constant (k3), which reflects metabolic trapping of FDG in hepatocytes, would be elevated in inflamed grafts, serving as a non-invasive biomarker for hepatic inflammation.

To this end, we launched the present pilot study, exploring the impact of liver transplantation on intrahepatic [^18^F]FDG kinetics through comparative analyses between LTx recipients and healthy control subjects. Additionally, we examined the feasibility of using dynamic [^18^F]FDG PET/CT to detect inflammation in asymptomatic LTx recipients. This assessment was conducted through comparative analyses between LTx recipients with biopsy-confirmed allograft inflammation and LTx recipients with histologically healthy grafts with no significant inflammation.

## 2. Materials and Methods

### 2.1. Study Design

Using a prospective recruitment strategy, sixteen clinically healthy LTx recipients were recruited and scanned using dynamic [^18^F]FDG imaging prior to a routine biopsy one year post-transplantation. Following their scan, subjects were biopsied within 2 weeks of the PET/CT scan, and their allograft was evaluated for signs of inflammation

Quantitative parameters, including SUVs (SUVmean, SUVmax) and compartmental kinetic rate constants (K1, k2, k3, and k4), were calculated from LTx recipients’ scans and compared with those of a separate healthy control group consisting of 8 individuals who underwent identical imaging protocols.

Histopathological evaluation of allograft inflammation was performed using the BANFF schema for grading liver allograft rejection [22]. LTx recipients with no significant allograft inflammation were compared to those of LTx recipients with a BANFF score of 3 or higher.

### 2.2. Patient Inclusion

Between February 2023 and May 2024, LTx recipients attending routine follow-ups at the outpatient liver transplant clinic at Copenhagen University Hospital Rigshospitalet were continuously screened for study eligibility (Study ID number: H-22068089). The inclusion criteria were liver transplantation ≥ 10 months prior to study entry, scheduled first routine biopsy since transplantation, and age ≥ 18 years.

LTx recipients were compared to a separate reference healthy control group recruited via online advertisements, who underwent identical imaging protocols (Study ID number: H-21034679). All control group participants were screened before inclusion in this study. Participants were eligible for the healthy control group if they had no history of liver transplantation, no chronic disease, no regular use of pharmaceuticals, and were aged ≥18 years.

Participants were excluded if they were pregnant, had claustrophobia, were unable to provide informed consent for psychological or other reasons, or were unable to read or speak Danish. Individuals with prematurely interrupted PET/CT scans were also excluded from the analyses.

### 2.3. PET/CT Scans

LTx recipients were scanned using a Biograph Vision Quadra LAFOV PET/CT scanner (Siemens Healthineers, Erlangen, Germany), a maximum of 2 weeks prior to the liver biopsy. Scans were performed in accordance with the most recent EANM guidelines for [^18^F]FDG use in inflammation assessment [23]; all participants fasted for 4 h, avoided strenuous exercise for 24 h, and had fasting blood sugar levels less than 11.1 mmol/L prior to the scan.

1 MBq/kg body-weight [^18^F]FDG was administered via a venous line placed in the left antecubital vein, followed by a dynamic PET acquisition in list-mode over 70 min spanning from vertex to mid-femur (collected into dynamic frames of 20 × 2 s, 10 × 5 s, 16 × 10 s, 6 × 60 s, 10 × 120 s, 8 × 300 s). Attenuation correction was performed using a low-dose CT scan (100 kVp, ref. mAs 30) without contrast enhancement.

PET images were reconstructed using an iterative 3D-OP-OSEM algorithm, including point spread function modelling and time-of-flight, with 4 iterations and 5 subsets. A Gaussian post-filter of 2 mm and a slice thickness of 2 mm were used. The image dimensions were a 440 × 440 voxel matrix with a 1.65 × 1.65 mm in-plane voxel size. Images were reconstructed using ultra-high-sensitivity mode with the full-acceptance angle geometry (maximum ring difference of 322).

SUVs were extracted from static PET images by positioning a spherical volume of interest (3 cm diameter) in the upper anterior part of the right liver lobe (segment 8) using the department’s routine clinical software, Syngo.Via (version VB80F, Siemens Healthineers, Erlangen, Germany).

The compartmental kinetic rate constants of FDG (K1, k2, k3, and k4) were calculated using an image-derived arterial input function [24], delay corrected to the mean activity curve of the liver as a blood concentration function. Kinetic rate constants were subsequently calculated using a three-compartment model (two-tissue compartment model).

### 2.4. Liver Biopsies

Within two weeks following PET imaging, two core-needle biopsies were obtained from the right liver lobe of each LTx recipient to ensure that representative tissue was extracted. Liver biopsies were processed, formalin-embedded, and cut in 2–4 µm thick sections, subsequently stained with HE, Masson’s trichrome, modified Sirius, iron, PAS, PAS with diastase, reticulin, oxidized orcein, CK7, C4d, and CD3. The biopsies were analyzed and scored according to BANFF guidelines for grading liver allograft rejections as illustrated in Supplementary Table S1 [22] BANFF score was calculated as a composite score ranging from 0 to 9, with points being assigned for portal inflammation (0–3 points), bile duct inflammation/damage (0–3 points), and endothelialitis (0–3 points).

LTx recipients were stratified into two groups based on BANFF score, having either significant inflammation on biopsy (BANFF Score ≥ 3, indicating borderline rejection or higher, requiring adjustment of immunosuppressant dosage and/or pulse steroid treatment) or no significant inflammation on biopsy (BANFF score < 3).

All biopsies were analyzed by a single pathologist (GLW), who was blind to [^18^F]FDG PET scan results, to avoid inter-observer variability and bias.

### 2.5. Collected Data Variables

The following data were collected for both LTx recipients and healthy controls: gender, age, weight, height, SUVmean, SUVmax, K1, k2, k3, and k4. For LTx recipients only, the following variables were collected: immunosuppressive treatment regimen, date of transplantation, date of liver biopsy, indication for transplantation, ALAT, HbA1c, portal inflammation score (0–3), bile duct inflammation score (0–3), endothelialitis score (0–3), and total BANFF score. Demographic data, transplant-related information, and biopsy results were obtained from national electronic databases. Kinetic parameters and SUVs were derived as described in Section 2.3.

### 2.6. Statistics and Analyses

Differences in scan results (SUVmean, SUVmax, K1, k2, k3, and k4) between the healthy control group and the LTx group were tested using Mann–Whitney U-tests. Differences were compared both including and excluding LTx recipients with significant inflammation on their biopsy (BANFF score of 3 or higher).

Due to the limited number of LTx recipients with significant inflammation in their biopsies (*n* = 3), statistical tests between the “BANFF Score ≥ 3” and “BANFFS Score < 3” groups were not performed. Instead, descriptive statistics including individual data points, data ranges, and medians are presented and visualized graphically.

Finally, scan results (SUVmean, SUVmax, K1, k2, k3 and k4) of LTx recipients were tested for correlation with BANFF score using Spearman’s rank correlation tests, with spearman rank coefficient values 0–0.19 indicating no correlation, values 0.2–0.39 indicating weak correlations, values 0.4–0.59 indicating moderate correlation, values 0.6–0.79 indicating strong correlations, values 0.8–0.99 indicating very strong correlations, and values of 1 indicating perfect correlation. Positive Spearman’s rank coefficient values indicate positive correlation between values, while negative rank coefficient values indicate inverse correlation.

Results with a *p*-value < 0.05 were considered statistically significant for all analyses. All statistical tests were conducted using RStudio (version 2024.12.10563).

## 3. Results

### 3.1. Patient Inclusion, Biopsy Results, and Baseline Characteristics

During the 15-month study period, 38 LTx recipients were screened for eligibility. Eight were excluded due to meeting one or more of the predefined exclusion criteria. Of the remaining 30 LTx recipients eligible for participation, five declined to participate, and an additional seven were unable to schedule a scan before their biopsy.

Of the 18 LTx recipients scanned, two procedures were interrupted at the patient’s request, resulting in 16 completed scans. All 16 LTx recipients underwent successful biopsy, and their data were included in the study. Additionally, eight healthy control participants were screened, scanned, and included; however, biopsies were not performed in this group. The final cohort thus comprised 16 LTx recipients (mean age 48.6 years; seven female, nine male) and eight healthy controls (mean age 35.4 years; six female, two male) (Table 1). No significant differences in gender, age, height, or weight were observed between the two groups.

At the time of imaging and biopsy, all 16 LTx recipients were receiving immunosuppressive treatment consisting of individualized doses of tacrolimus, everolimus, or cyclosporine administered once or twice daily, supplemented with mycophenolate mofetil, mycophenolate sodium, and/or prednisone. No two patients received identical immunosuppressive regimens.

**Figure 2:**
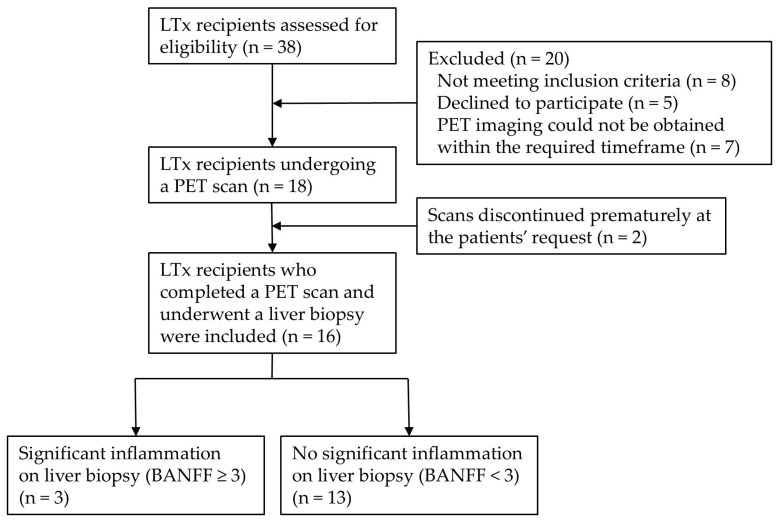
Consort diagram of liver transplant (LTx) recipient recruitment process.

Of the 16 LTx recipients, 13 had no significant inflammation on their biopsy (BANFF Score < 3). Three LTx recipients had BANFF scores ≥ 3; two had a score of three, and one had a score of six. All three LTx recipients with BANFF scores ≥ 3 were treated with an increase in regular immunosuppressant dosage and/or pulse immunosuppressant treatment. The three LTx recipients with BANFF scores ≥ 3 were subsequently re-biopsied within 3 months. All three re-biopsies were scored as BANNF < 3, showing remission of inflammation. A consort diagram summarizing the LTx recipient recruitment process and stratification based on biopsy results can be found in Figure 2, and baseline characteristics can be found in Table 1 below:

### 3.2. Differences Between Control Group and LTx Recipients

Since there were only three patients with significant inflammation (i.e., a BANFF score greater than three), these patients were not compared to the healthy controls.

Dynamic PET analysis showed a significantly higher mean k3 value in LTx recipients (k3 = 0.0053 min^−1^ ± 0.006 min^−1^) than in healthy controls (k3 = 0.0037 min^−1^ ± 0.0003 min^−1^) (*p* = 0.0159). The difference in k3 between LTx recipients and healthy controls was still present in subgroup analyses excluding LTx recipients with significant inflammation on their biopsy (k3 = 0.0050 min^−1^ ± 0.0004 min^−1^) (*p* = 0.0099).

Similarly, mean k4 values were also significantly higher in LTx recipients (k4 = 0.0061 min^−1^ ± 0.0016 min^−1^) than in healthy controls (k4 = 0.0019 min^−1^ ± 0.0011 min^−1^) (*p* = 0.0382). The difference in k4 between LTx recipients and healthy controls was also present in subgroup analyses excluding LTx recipients with significant inflammation on their biopsy (k4 = 0.0070 min^−1^ ± 0.0019 min^−1^) (*p* = 0.0199).

**Figure 3:**
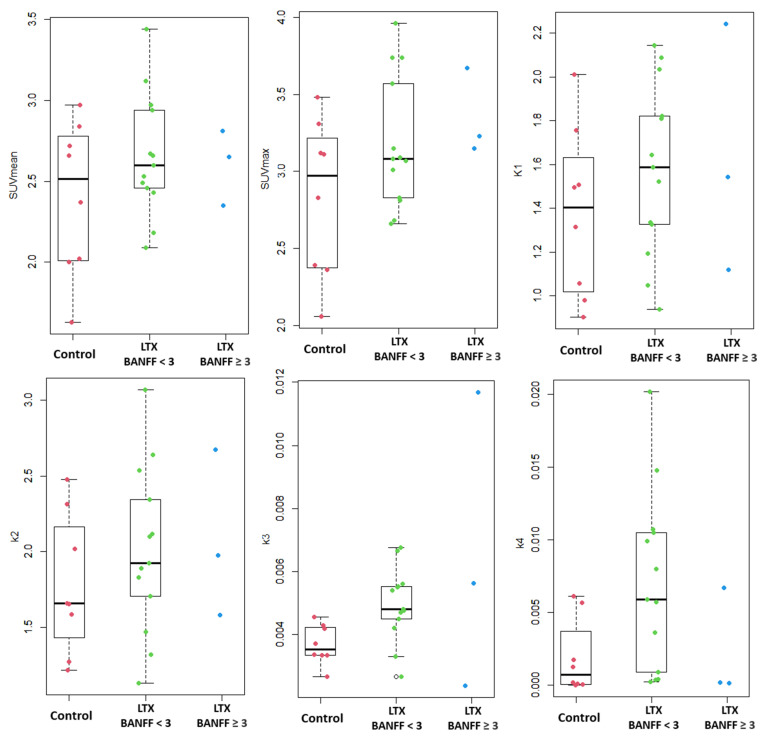
Boxplot visualization of kinetic parameters and SUVs in the control group, as well as the liver transplant (LTx) group, stratified by BANFF score at liver biopsy. Only individual datapoint values are shown in the “BANFF score ≥ 3” group, as there are not enough data-points available to produce a meaningful boxplot (*n* = 3).

No significant differences in SUVmean, SUVmax, K1, or k2 were found between groups. Group means values with standard deviations (SDs) for SUVs and kinetic rate constants are summarized in Table 2 and depicted by boxplots in Figure 3 below:

### 3.3. Comparing LTx Recipients with and Without Inflammation

As only three patients had significant inflammation on biopsy, statistical tests for significance comparing LTx recipients with and without inflammation were not performed.

**Figure 4:**
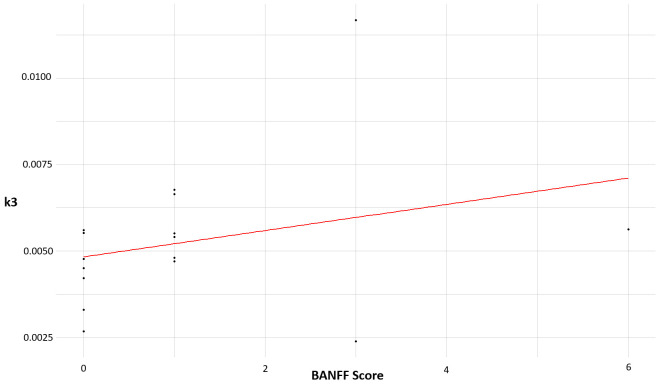
Relationship between BANFF score and k3. Plot of the relationship between BANFF score and k_3_ in transplant recipients with linear trendline (red colour).

Descriptive analyses of kinetic rate constants revealed no consistent pattern or differences between LTx recipients with significant inflammation (BANFF score ≥ 3) and LTx recipients without significant inflammation (BANFF score < 3). Among the three LTx recipients with significant inflammation, one LTx recipient with a BANFF score of 3 had a k3 value (k3 = 0.0117 min^−1^) which was markedly higher than those observed in LTx recipients with BANFF scores < 3 (range: 0.0027 min^−1^ to 0.0068 min^−1^). However, the remaining two with BANFF scores ≥ 3 had k3 values like those with BANFF scores < 3; the LTx recipient with a BANFF score of three had a k3 value of 0.0024, and the LTx recipient with a BANFF score of six had a k3 value of 0.0056. The relationship between k3 and the BANFF score in LTx recipients is visualized in Figure 4.

No convincing differences were observed in SUVmean, SUVmax, K1, k2, or k4 between LTx recipients with and without significant inflammation. Result ranges, medians, and individual datapoints are visualized in Figure 3.

Spearman rank correlation tests showed no significant correlations between BANFF score and any of the tested scan results (SUVmean, SUVmax, K1, k2, k3, or k4). Spearman’s rank coefficients are listed in Table 3. The strongest degree of correlation was found between BANFF score and k3, indicating a weak positive but non-significant association (k3: rs = 0.396, *p* = 0.128).

## 4. Discussion

This pilot study of dynamic LAFOV [^18^F]FDG PET/CT in liver transplant recipients demonstrated significantly higher k3 and k4 values compared with healthy controls, but did not show a significant correlation between inflammation (as measured by the BANFF score) and any kinetic parameter or standardized uptake value.

The observation that LTx recipients exhibited higher k3 and k4 values than healthy controls, regardless of the inclusion of patients with BANFF scores ≥ 3, suggests that fundamental differences may exist in FDG kinetics between liver allografts and non-transplant livers, even in the absence of rejection-associated inflammation. In particular, insulin resistance may alter tracer dynamics independently of inflammation. Previous studies have suggested that insulin resistance can increase k3 and k4 values under fasting conditions, potentially reflecting altered glucose transport and phosphorylation [25]. Immunosuppressive agents commonly used in transplant recipients, including tacrolimus and cyclosporine, have been associated with the development of insulin resistance and hyperinsulinemia [26,27,28]. As all LTx recipients in this study were receiving such therapies at the time of imaging, drug-induced metabolic alterations may have contributed to the observed elevation in kinetic parameters. Importantly, the relatively normal mean HbA1c levels in this cohort suggest that overt dysglycemia was not present [29]; however, HbA1c may not fully capture subtle or early insulin resistance. In addition to insulin resistance, other post-transplant metabolic changes—such as altered hepatic perfusion, changes in cellular composition of the graft, or systemic inflammatory effects of immunosuppression—may further influence FDG uptake and kinetics. Collectively, these factors may confound the relationship between PET-derived parameters and true inflammatory activity, thereby limiting the specificity of dynamic [^18^F]FDG PET/CT for detecting rejection-associated inflammation in liver allografts. Given the lack of a clear pattern between scan results and the presence of inflammation, this study does not provide convincing evidence that [^18^F]FDG PET scans are a suitable alternative to routine biopsies in identifying LTx recipients in need of additional immunosuppressants. To be able to use PET instead of biopsy to guide immunosuppressive treatment, high test sensitivity would be required in order to identify rejection cases, where higher amounts of immunosuppression are required. A high specificity is also required to avoid unnecessary administration of too much immunosuppressant due to false positives. The large spread of measurements amongst LTx recipients with BANFF scores ≥ 3, as well as the similarities to the measurements from LTx recipients with BANFF scores < 3, do not bode well for the probability of achieving a satisfactory sensitivity and specificity. However, it should be kept in mind that this study is a pilot study, and that the number of LTx recipients with significant inflammation was very small in the present study (*n* = 3). Definitive conclusions should therefore not be drawn from these data.

It should also be noted that this study classifies BANFF scores of three, an amount of inflammation indicating suspicion of borderline rejection, as having significant inflammation. In fact, two out of three LTx recipients in this group only had a BANFF score of three. It might be that this relatively low amount of inflammation is not enough to induce a detectable difference in FDG kinetics. It is possible that larger studies including LTx recipients with more pronounced amounts of inflammation/rejection would produce different results. However, in order to be able to be used as an effective alternative to routine biopsies, even small amounts of inflammation would have to be detectable.

Strengths of this study include the consistent methodology employed throughout the study: All study participants were scanned and biopsied using identical techniques and equipment, and all analyses were conducted by the same persons, eliminating most major sources of bias. Furthermore, the study was conducted using state-of-the-art PET imaging equipment, enhancing the accuracy of the data.

Limitations of this study stem primarily from the small sample size of included participants, especially in the subgroup of LTx recipients with significant inflammation on their biopsy (*n* = 3). Furthermore, although there were no significant differences in age or weight between healthy controls and transplant recipients, healthy controls trended towards being younger and weighing less, which could potentially skew results.

The small sample size makes parametric testing unreliable, hence the use of non-parametric statistical tests. It should also be noted that no tests of significance were performed for differences between LTx recipients with and without inflammation, and that these two groups were only compared descriptively. Additionally, due to the small sample size, no tests of diagnostic performance were performed. To draw definitive and clinically applicable conclusions regarding the viability of [^18^F]FDG PET/CT for diagnosing inflammation in LTx recipients, larger studies incorporating sensitivity and specificity analyses, as well as receiver operating characteristic (ROC) analyses, are required.

## 5. Conclusions

The 16 liver transplant recipients exhibited significantly higher mean k3 and k4 values than the eight healthy controls, indicating increased FDG phosphorylation and dephosphorylation. These differences were not explained by rejection-associated inflammation, suggesting fundamental alterations in FDG kinetics in liver allografts compared with native livers. This may represent a potential confounder in [^18^F]FDG PET/CT, possibly related to insulin resistance or other immunosuppression-induced metabolic changes.

No significant correlation was observed between any imaging parameters (SUVmean, SUVmax, K1, k2, k3, or k4) and BANFF score. Although descriptive analyses of the imaging results in three liver transplant recipients with biopsy-confirmed significant inflammation did not demonstrate clear differences compared with the 13 recipients without significant inflammation, the small number of affected patients precludes definitive conclusions regarding diagnostic performance or clinical applicability. Larger studies incorporating parametric analyses, as well as sensitivity, specificity, and ROC analyses, are needed to determine whether dynamic LAFOV PET/CT can be used as an alternative to routine biopsies for diagnosing inflammation in transplanted livers.

## Figures and Tables

**Table 1:** Baseline characteristics of the healthy control group and liver transplant (LTx) group. * *p*-value calculated using the Mann–Whitney U-test. ** “Other” includes non-alcoholic steatohepatitis, primary biliary cholangitis, hepatic metastases, and autoimmune hepatitis.

	Healthy Control Group (*n* = 8)	LTx Group (*n* = 16)	*p*-Value * LTx Group vs. Control Group
Gender (*n*)—F: female, M: male	F: 6, M: 2	F: 7, M: 9	0.21
Age (years)—mean	35.4	48.6	0.081
[range]	[22–67]	[19–67]	
Height (cm)—mean	172	175	0.358
[range]	[160–188]	[161–192]	
Weight (kg)—mean	71	81	0.374
[range]	[48–83]	[54–115]	
Reason for LTX (*n*)			
Hepatocellular carcinoma	-	3	-
Alcoholic Cirrhosis	-	4	-
Primary sclerosing cholangitis	-	4	-
Other **	-	5	-
Inflammation on Biopsy (*n*)			
BANFF < 3	-	13	-
BANFF ≥ 3	-	3	-
Immunosuppressant used (*n*)			
Tacrolimus		14	
Everolimus		1	
Cyclosporine		1	
Mycophenolate Mofetil		11	
Mycophenolate Sodium		2	
Prednisone		14	
ALAT (U/L)—mean	-	37	-
[Range]		[9–234]	
HbA1c (mmol/mol)—mean	-	38	-
[Range]		[26–54]	

**Table 2:** Comparison of kinetic parameters and standardized uptake values (SUVs) between the control group and liver transplant (LTx) group. BANFF < 3 represents LTx recipients without significant inflammation on their liver biopsy. Values are presented as group means (±standard deviation). * Calculated using the Mann–Whitney U-test. ** Significant difference at *p* < 0.05.

	Control Group (*n* = 8)	All LTx Recipients (*n* = 16)	*p*-Value * All LTx Recipients vs. Control Group	LTx Recipients with BANFF < 3 (*n* = 13)	*p*-Value * LTx Recipients with BANFF < 3 vs. Control Group
SUVmean	2.40 ± 0.20	2.65 ± 0.09	0.374	2.66 ± 0.12	0.328
SUVmax	2.83 ± 0.21	3.22 ± 0.11	0.178	3.18 ± 0.13	0.310
K1	1.38 ± 0.16	1.59 ± 0.11	0.172	1.58 ± 0.17	0.210
k2	1.78 ± 0.20	2.02 ± 0.14	0.291	2.01 ± 0.17	0.301
k3	0.0037 ± 0.0003	0.0053 ± 0.006	0.0159 **	0.0050 ± 0.0004	0.0099 **
k4	0.0019 ± 0.0011	0.0061 ± 0.0016	0.0382 **	0.0070 ± 0.0019	0.0199 **

**Table 3:** Correlation analyses between PET-derived parameters and BANFF score in LTx recipients. Spearman’s rank correlation test is applied. An r_s_ value of 1 indicates perfect correlation. An r_s_ value of −1 indicates perfect inverse correlation.

	Spearman’s Rank Correlation Coefficient (r_s_)	*p*-Value for Correlation with BANFF Score
SUVmean	−0.109	0.687
SUVmax	0.124	0.649
K1	−0.014	0.958
k2	0.044	0.871
k3	0.396	0.128
k4	−0.117	0.666

## Data Availability

Deidentified datasets generated and analyzed during the current study are available from the corresponding author on reasonable request from qualified scientific and medical researchers. Due to local national data protection laws, all requests will be reviewed by the author group and data provided according to conditions laid by the author group, as all data transfers require approval from the Danish Data Protection Agency and the Ethics Committee in the Capital Region of Denmark.

## Supplementary Materials

The following supporting information can be downloaded at: https://www.mdpi.com/article/10.3390/diagnostics16071021/s1, Table S1: BANFF schema for grading liver allograft inflammation.

## Abbreviations

The following abbreviations are used in this manuscript:

[^18^F]FDG	2-[^18^F]fluoro-2-deoxy-D-glucose
PET/CT	Positron Emission Tomography/Computerized Tomography
SUV	Standardized Uptake Value
LAFOV	Long-Axial Field of View
SAFOV	Short-Axial Field of View
LTx	Liver Transplant
