# Supplementary material for: Pilot Study on Dynamic Long-Axial Field-of-View [18F]FDG PET/CT in Liver Transplant Recipients as a Non-Invasive Alternative to Routine Biopsies
Source: Diagnostics (Basel). 2026 Mar 28;16(7):1021. doi: 10.3390/diagnostics16071021 (PMC13073691; doi:10.3390/diagnostics16071021)
Supplement: Supplementary file 1 [file diagnostics-16-01021-s001.zip › diagnostics-4191427-supplementary.pdf]

Parameter	Histological Presentation	Score
**Portal Inflammation**	Mostly lymphocytic inflammation involving, but not noticeably expanding, a minority of the triads	1
	Expansion of most or all triads, by a mixed infiltrate containing lymphocytes with occasional blasts, neutrophils, and eosinophils	2
	Marked expansion of most or all triads by a mixed infiltrate containing numerous blasts and eosinophils with inflammatory spillover into the periportal parenchyma	3
**Bile Duct Inflammation/ Damage**	A minority of the ducts are cuffed and infiltrated by inflammatory cells and show only mild reactive changes, such as increased nuclear:cytoplasmic ratio of the epithelial cells	1
	Most or all ducts are infiltrated by inflammatory cells. More than an occasional duct shows degenerative changes such as nuclear pleomorphism, disordered polarity, and cytoplasmic vacuolization of the epithelium	2
	As above, with most or all ducts showing degenerative changes or focal luminal disruption	3
**Endothelialitis**	Subendothelial lymphocytic infiltration involving some, but not a majority of the portal and/or hepatic venules	1
	Subendothelial infiltration involving most or all portal and/or hepatic venules	2
	As above, with moderate or severe perivenular inflammation that extends into the perivenular parenchyma and is associated with perivenular hepatocyte necrosis	3

Supplementary Table S1. BANFF schema for grading liver allograft inflammation—adopted from *Banff schema for grading liver allograft rejection: An international consensus document* by Demetris A. J et al. (22).

Composite BANFF score thresholds:

- **0–2: No rejection / No significant inflammation**
- **3: Borderline rejection**
- **4–5: Mild rejection**
- **6–7: Moderate rejection**
- **8–9: Severe rejection**
